# Infection risk associated with talquetamab in relapsed/refractory multiple myeloma: a systematic review with meta-analysis of talquetamab monotherapy and descriptive analysis of combination therapy

**DOI:** 10.3389/fimmu.2026.1848518

**Published:** 2026-08-19

**Authors:** Zeng-Yi Huang, Weilong Wu, Gaoyu Luo, Hui Huang, Xiao-Lian Liu

**Affiliations:** 1 Department of Hematology, Gaozhou People’s Hospital, Maoming, Guangdong, China; 2 Department of Dermatology, The First Affiliated Hospital, Jinan University, Guangzhou, China

**Keywords:** bispecific antibodies, infection, meta-analysis, multiple myeloma, real-world evidence, talquetamab

## Abstract

**Background:**

Talquetamab, a first-in-class G protein–coupled receptor family C group 5 member D (GPRC5D) × CD3 bispecific antibody, demonstrates substantial clinical activity in relapsed/refractory multiple myeloma (RRMM). However, immune dysfunction associated with advanced disease, extensive prior therapies, and bispecific antibody–mediated immune modulation may increase susceptibility to infections. This systematic review and meta-analysis aimed to characterize infection risk associated with talquetamab, with separate evaluation of monotherapy and combination therapy settings.

**Methods:**

PubMed, Embase, Web of Science Core Collection, and Cochrane Library databases were searched from inception to July 1, 2026. Studies reporting infection outcomes among RRMM patients treated with talquetamab-containing regimens were eligible. Outcomes included any-grade infections, grade ≥3 infections, infection-related mortality, reported pathogens, and preventive strategies when available. Because talquetamab monotherapy and combination regimens represent clinically distinct treatment strategies, quantitative analyses were restricted to monotherapy cohorts, whereas combination therapy studies were summarized descriptively. A random-effects model was used to estimate pooled infection incidence. Methodological quality was assessed using the Joanna Briggs Institute (JBI) Critical Appraisal Checklist for Case Series.

**Results:**

Six studies were included in the qualitative synthesis, including four talquetamab monotherapy cohorts and two talquetamab-based combination therapy cohorts. Quantitative meta-analysis included four monotherapy cohorts comprising 735 patients for any-grade infections and three cohorts comprising 584 patients for grade ≥3 infections. The pooled incidence of any-grade infections was 51% (95% CI, 34%–67%; I² = 94.2%), while the pooled incidence of grade ≥3 infections was 22% (95% CI, 17%–28%; I² = 59.6%). Given the limited number of available studies and substantial heterogeneity, these pooled estimates should be interpreted as exploratory and hypothesis-generating. Combination therapy cohorts, including talquetamab plus teclistamab and talquetamab-based regimens from MonumenTAL-3, demonstrated substantial infection burdens and were characterized descriptively rather than quantitatively. Infection-related mortality ranged from 0% to 3.2%. Frequently reported infections included viral infections (particularly SARS-CoV-2 and cytomegalovirus), bacterial pneumonia, and opportunistic infections such as Pneumocystis jirovecii pneumonia.

**Conclusions:**

Talquetamab monotherapy in heavily pretreated RRMM patients is associated with a clinically meaningful risk of infections, including severe infections. The substantial heterogeneity observed across studies likely reflects differences in patient characteristics, prior therapies, treatment settings, infection definitions, and supportive care strategies. Combination bispecific antibody regimens may represent a potentially higher infection burden requiring enhanced infection surveillance and preventive approaches. Further prospective studies are warranted to better define infection risk factors and optimal prevention and management strategies during talquetamab therapy.

**Systematic review registration:**

https://www.crd.york.ac.uk/prospero/display_record.php?ID=CRD420261357352, identifier CRD420261357352.

## 1 Introduction

The therapeutic landscape of relapsed/refractory multiple myeloma (RRMM) has been substantially transformed by T-cell–redirecting immunotherapies, particularly bispecific antibodies targeting novel plasma cell antigens (1, 2). Among these, talquetamab, a first-in-class G protein–coupled receptor family C group 5 member D (GPRC5D) × CD3 bispecific antibody, has demonstrated clinically meaningful activity in heavily pretreated patients, including those previously exposed to B-cell maturation antigen (BCMA)-directed therapies (3, 4). GPRC5D is primarily expressed on malignant plasma cells and certain keratinized tissues and is largely absent from early B-cell lineage precursors (5). These biological characteristics suggest that talquetamab may have a distinct immunologic safety profile compared with BCMA-directed agents; nevertheless, clinically significant infections remain an important concern during treatment (6, 7). This increased susceptibility to infection may reflect sustained T-cell activation, treatment-related cytopenias, cumulative exposure to immune-directed therapies, and the underlying immune dysfunction characteristic of heavily pretreated RRMM populations (8). In addition, the emerging use of combination bispecific antibody strategies may further complicate infection risk profiles (9, 10).

Although pivotal clinical trials provide valuable information on safety outcomes under controlled conditions, they may not fully reflect routine clinical practice because of differences in patient characteristics, prior treatment exposure, supportive care strategies, and follow-up duration. Real-world evidence can therefore complement clinical trial data by providing additional insights into infection risk and management patterns across diverse clinical settings (11–13).

Available real-world studies have reported substantial variability in infection incidence and severity, while pathogen-specific outcomes and preventive strategies have been reported inconsistently (13, 14).

Although several meta-analyses have evaluated the overall safety profiles of bispecific antibodies in multiple myeloma (15, 16), a dedicated synthesis focusing specifically on infection risk associated with talquetamab has not yet been reported. Therefore, we conducted this systematic review and meta-analysis to (1) estimate the pooled incidence of any-grade and grade ≥3 infections associated with talquetamab monotherapy; (2) characterize infection patterns, infection-related mortality, reported pathogens, and preventive strategies across talquetamab-treated populations; and (3) descriptively summarize infection outcomes associated with emerging talquetamab-based combination regimens.

## 2 Methods

### 2.1 Study design and registration

This systematic review and meta-analysis was conducted in accordance with the Preferred Reporting Items for Systematic Reviews and Meta-Analyses (PRISMA 2020) guidelines (17). The study protocol was prospectively registered in the PROSPERO database (registration number: CRD420261357352) (18).

### 2.2 Literature search strategy

A comprehensive literature search was performed in PubMed (MEDLINE), Embase, Web of Science Core Collection, and the Cochrane Library from inception to July 1, 2026.

The search strategy was developed to identify studies investigating talquetamab and infection-related outcomes. The search combined controlled vocabulary terms and free-text keywords related to:

1. talquetamab or its alternative identifiers, including GPRC5D, JNJ-64407564, JNJ64407564, and Talvey;
2. infection-related outcomes, including infection, infectious disease, pneumonia, sepsis, viral infection, bacterial infection, fungal infection, opportunistic infection, COVID-19, and adverse events.

The detailed database-specific search strategies are provided in **Supplementary Table 2**.

No restrictions were applied regarding publication status; however, only English-language publications were eligible. Reference lists of relevant articles were manually screened to identify additional potentially eligible studies. Potentially overlapping publications were assessed by comparing study populations, enrollment periods, institutional sources, and reported sample characteristics. When multiple reports derived from the same cohort were identified, the most comprehensive or latest report providing extractable infection-related outcomes was selected.

### 2.3 Eligibility criteria

Studies were included if they met the following criteria:

1. adult patients diagnosed with relapsed or refractory multiple myeloma (RRMM);
2. treatment with talquetamab as monotherapy or as part of a combination regimen;
3. reported infection-related outcomes, including any-grade infections, grade ≥3 infections, infection-related mortality, or specific pathogens;
4. prospective clinical trials or real-world observational studies.

For quantitative synthesis, only studies evaluating talquetamab monotherapy were included to minimize clinical heterogeneity. Studies evaluating talquetamab-based combination regimens were retained for qualitative synthesis and descriptive analysis only. Potentially overlapping cohorts from different publications were assessed individually, and duplicate or companion reports were excluded from quantitative synthesis.

Studies were excluded if they were:

1. reviews, editorials, narrative articles, case reports, or preclinical studies;
2. conference abstracts without sufficient extractable infection data;
3. duplicate reports or overlapping patient cohorts;
4. studies lacking separately extractable infection outcomes associated with talquetamab treatment.

### 2.4 Study selection and data extraction

Two reviewers independently screened titles and abstracts, followed by full-text assessment of potentially eligible studies. Discrepancies were resolved through discussion or consultation with a third reviewer.

The following data were extracted from each included study: study characteristics (first author, publication year, study design, sample size, and treatment regimen), baseline patient characteristics, and infection-related outcomes, including any-grade infections, grade ≥3 infections, infection-related mortality, reported pathogens, and available supportive-care measures (e.g., intravenous immunoglobulin use and antimicrobial prophylaxis and vaccination). For each infection outcome, the corresponding evaluable population and denominator were recorded when available.

Treatment regimens were categorized as talquetamab monotherapy or talquetamab-based combination therapy. Infection outcomes from combination therapy cohorts were extracted and summarized descriptively but were not included in the primary quantitative meta-analysis. Infection severity was extracted according to the grading criteria reported in each study. When available, the corresponding grading systems and CTCAE versions were recorded. The dedicated MonumenTAL-1 infection analysis used CTCAE version 4.03 for infection grading. For publications reporting potentially overlapping cohorts, eligibility was determined after review of cohort characteristics, including recruitment period, study population, and treatment exposure. Only non-overlapping or most comprehensive datasets were included in the final synthesis.

### 2.5 Quality assessment

The methodological quality of included studies was assessed using the Joanna Briggs Institute (JBI) Critical Appraisal Checklist for Case Series (19). As all quantitative analyses were based on uncontrolled single-arm cohorts without comparator groups for infection outcome assessment, the JBI case-series checklist was considered appropriate for evaluating methodological limitations related to single-arm safety reporting. Each study was assessed across ten domains, including clear inclusion criteria, reliable outcome measurement, and appropriate statistical analysis.

### 2.6 Statistical analysis

Meta-analysis of infection proportions was performed using a random-effects model to account for between-study variability. Quantitative synthesis was restricted to talquetamab monotherapy cohorts. Proportions were transformed using the Freeman–Tukey double arcsine transformation to stabilize variances before pooling, following established methods for meta-analysis of proportions (20). Pooled incidences of any-grade and grade ≥3 infections were calculated with corresponding 95% confidence intervals (CIs). Prediction intervals were calculated when sufficient studies were available.

Combination therapy studies were summarized descriptively and were not included in pooled estimates because of potential differences in treatment intensity and immune effects. Statistical heterogeneity was assessed using the I² statistic and Cochran’s Q test. Sensitivity analyses were performed using a leave-one-out approach to evaluate the robustness of pooled estimates.

All statistical analyses were performed using R software (version 4.3.1; R Foundation for Statistical Computing, Vienna, Austria) with the “meta” package (21).

## 3 Results

### 3.1 Study selection

The database search identified 416 records, including 52 from PubMed, 319 from Embase, 31 from Web of Science Core Collection, and 14 from the Cochrane Library. After removal of 180 duplicate records, 236 records were screened based on titles and abstracts. Of these, 212 records were excluded because they were unrelated to talquetamab treatment, did not report infection outcomes, or represented inappropriate publication types.

A total of 24 reports were retrieved for full-text assessment. Among these, 18 reports were excluded due to reasons including review/editorial articles or other non-original publications (n = 9), lack of extractable infection-related outcomes (n = 2), preclinical studies or case reports (n = 3), overlapping cohort/companion reports (n = 1), and inappropriate population, intervention, or non-separable infection data (n = 3).

**Figure 1:**
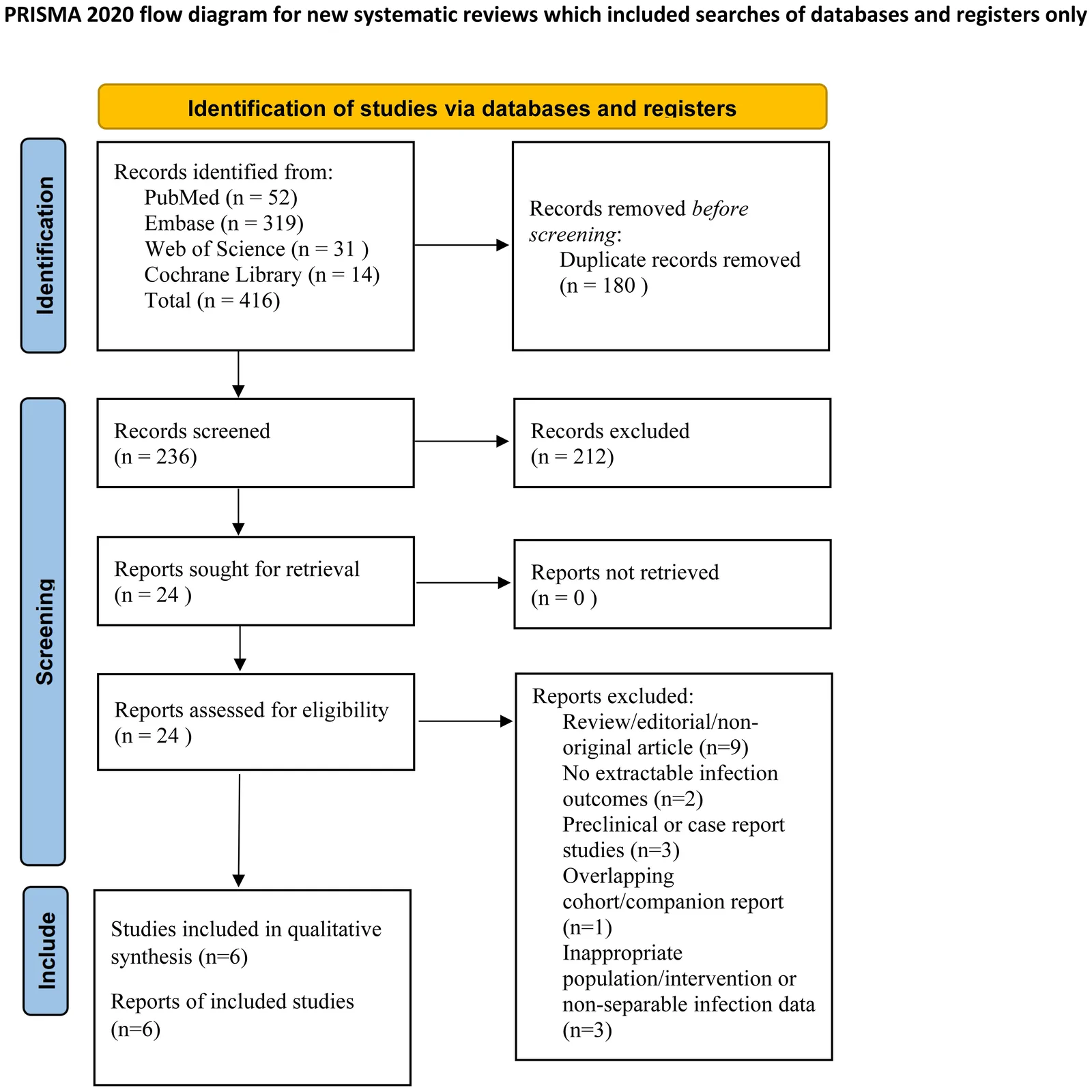
PRISMA 2020 flow diagram of the study selection process. The diagram illustrates the identification, screening, eligibility assessment, and inclusion process of studies according to the PRISMA 2020 guidelines. A total of 416 records were identified from four electronic databases (PubMed, Embase, Web of Science Core Collection, and Cochrane Library). After removal of duplicates and title/abstract screening, 24 reports underwent full-text assessment. Six studies were included in the qualitative synthesis, including four talquetamab monotherapy cohorts and two talquetamab-based combination therapy cohorts. Among these, four talquetamab monotherapy cohorts provided extractable infection outcomes and were included in quantitative analyses, whereas talquetamab-based combination therapy studies were retained for descriptive synthesis only because of potential differences in treatment strategies, immune effects, and infection risk profiles.

Following assessment of eligibility and potential cohort overlap, six studies were included in the qualitative synthesis (9, 11, 12, 22–24). These included four talquetamab monotherapy cohorts derived from MonumenTAL-1 (22, 25), Frenking et al. (12), Al Hadidi et al. (11), and Janakiram et al. (23) and two talquetamab-based combination therapy cohorts (RedirecTT-1 (9) and MonumenTAL-3 (24)). For MonumenTAL-1, baseline study characteristics were obtained from the primary trial publication (22), whereas infection outcomes used in the quantitative meta-analysis were extracted from the dedicated infection analysis report (25). The four monotherapy cohorts contributed data to the quantitative meta-analysis, whereas the two combination therapy studies were retained for descriptive analysis only because of differences in treatment strategies and potential clinical heterogeneity. The study selection process is summarized in the PRISMA flow diagram (**Figure 1**).

### 3.2 Study characteristics and quality assessment

The six included studies evaluated talquetamab-containing regimens in patients with relapsed/refractory multiple myeloma (RRMM), and their baseline characteristics are summarized in **Table 1**. Among these, four studies evaluated talquetamab monotherapy, including one prospective clinical trial (MonumenTAL-1) (22) and three retrospective real-world evidence studies (Frenking et al. (12), Al Hadidi et al. (11), and Janakiram et al. (23)). Two additional prospective studies investigated talquetamab-based combination regimens: RedirecTT-1 evaluated talquetamab plus teclistamab (9), whereas MonumenTAL-3 evaluated talquetamab-based therapy combined with daratumumab with or without pomalidomide (24). These combination therapy studies were analyzed separately because they represent distinct treatment strategies with potentially different degrees of immune modulation and infection risk profiles.

**Table 1:** Characteristics of included studies and baseline patient characteristics.

Study (year)	Study type	Treatment regimen	N	Median age, years (range)	Median prior lines (range)	Triple-class refractory, n/N (%)	Prior BCMA-directed therapy, n/N (%)	High-risk cytogenetics, n/N (%)
MonumenTAL-1 (2025)	Phase 1–2 trial	Talquetamab monotherapy	375	67 (33–86)	5 (2–17)	267/375 (71.3%)	113/375 (30.2%)	91/375 (24.3%)
Al Hadidi et al. (2025) (11)	Multicenter retrospective RWE, US	Talquetamab monotherapy	114	67 (34–90)	6 (2–15)	114/114 (100%)	74/114 (64.9%)	64/91 (70.3%)
Frenking et al. (2025) (12)	Multicenter retrospective RWE, Germany	Talquetamab monotherapy	138	69 (40–88)	6 (2–15)	118/138 (85.5%)	NR‡	57/120 (47.5%)
RedirecTT-1 (2025)	Phase 1b–2 trial	Talquetamab + teclistamab	94	64.5 (39–82)	4 (1–11)	81/94 (86.2%)	NR†	21/51 (41.2%)
Janakiram et al. (2026) (23)	Multicenter retrospective RWE, international IMWG registry	Talquetamab monotherapy	151	64 (35–86)	6 (1–20)	137/151 (91.3%)	113/151 (74.8%)	43/103 (41.7%)
MonumenTAL-3 (2026)	Phase 3 randomized trial	Talquetamab + daratumumab ± pomalidomide	574	64 (30–88)	2 (1–8)	NR	NR	185/574 (32.2%)

BCMA, B-cell maturation antigen; BTCE, bispecific T-cell engager; CAR-T, chimeric antigen receptor T-cell therapy; IMWG, International Myeloma Working Group; NR, not reported; RCT, randomized controlled trial; RWE, real-world evidence; TCR, T-cell redirection therapy.

Baseline characteristics were extracted from the original reports. For MonumenTAL-1, baseline characteristics were obtained from the updated primary trial report including 375 patients, whereas infection outcomes were extracted from the dedicated infection analysis report including 339 evaluable patients. Therefore, baseline characteristics and infection outcomes were derived from different analytic populations.

For MonumenTAL-3, baseline characteristics were extracted from the talquetamab-containing treatment groups (Tal-DP and Tal-D; n=574) of the intention-to-treat population. Infection outcomes were assessed in the safety population of patients receiving talquetamab-containing regimens and were summarized descriptively. The study was not included in quantitative pooled analyses because of differences in treatment strategies and potential clinical heterogeneity compared with talquetamab monotherapy cohorts.

Janakiram et al. represented an international IMWG Immunotherapy Registry real-world cohort of patients treated with talquetamab monotherapy.

For Frenking et al., baseline characteristics were extracted from the overall cohort of 138 patients, whereas infection outcomes were available for 131 evaluable patients.

Percentages for high-risk cytogenetics were calculated among patients with available cytogenetic data.

^†^In RedirecTT-1, prior BCMA-directed therapy was not separately reported because previous BCMA-directed treatment categories were not mutually exclusive.

^‡^Frenking et al. reported prior bispecific T-cell engager exposure and CAR-T exposure separately; these categories may overlap, and a non-overlapping overall proportion of prior BCMA-directed therapy was not available.

Summary of study design, treatment characteristics, and baseline clinical features of included cohorts. The table presents study type, treatment regimen, sample size, age distribution, prior treatment exposure, triple-class refractory status, prior BCMA-directed therapy exposure, and high-risk cytogenetic characteristics. Included patients were predominantly heavily pretreated individuals with relapsed/refractory multiple myeloma. Baseline characteristics and infection outcomes were extracted from the corresponding original reports. For MonumenTAL-1, baseline characteristics were obtained from the updated primary trial report, whereas infection outcomes were extracted from the dedicated infection analysis cohort. Talquetamab-based combination therapy studies (RedirecTT-1 and MonumenTAL-3) were included for descriptive assessment only and were not included in quantitative pooling.

For MonumenTAL-1, baseline characteristics were extracted from the updated primary trial report (22), whereas infection-related outcomes were obtained from the dedicated infection analysis report (25). Therefore, the denominators for baseline characteristics and infection outcomes were not identical in this cohort.

Across the included studies, patients were generally heavily pretreated, with a median of 4–6 prior lines of therapy (9, 11, 12, 22–24). Triple-class refractory disease was frequently reported, particularly among real-world cohorts, and prior exposure to BCMA-directed therapies was common (9, 11, 12, 22–24). These findings indicate that the available evidence primarily represents a population with advanced RRMM and substantial underlying immune dysfunction.

Methodological quality assessment was performed for the four talquetamab monotherapy cohorts included in the quantitative synthesis using the Joanna Briggs Institute (JBI) Critical Appraisal Checklist for Case Series. Overall, the included cohorts demonstrated generally acceptable methodological quality, with most domains rated as “Yes”. Several domains were judged as “Unclear”, mainly because of incomplete reporting of participant selection details, study setting, or follow-up information (**Supplementary Table 3**, **Supplementary Figure 1**).

### 3.3 Primary outcomes: infection risk associated with talquetamab monotherapy

**Figure 2:**
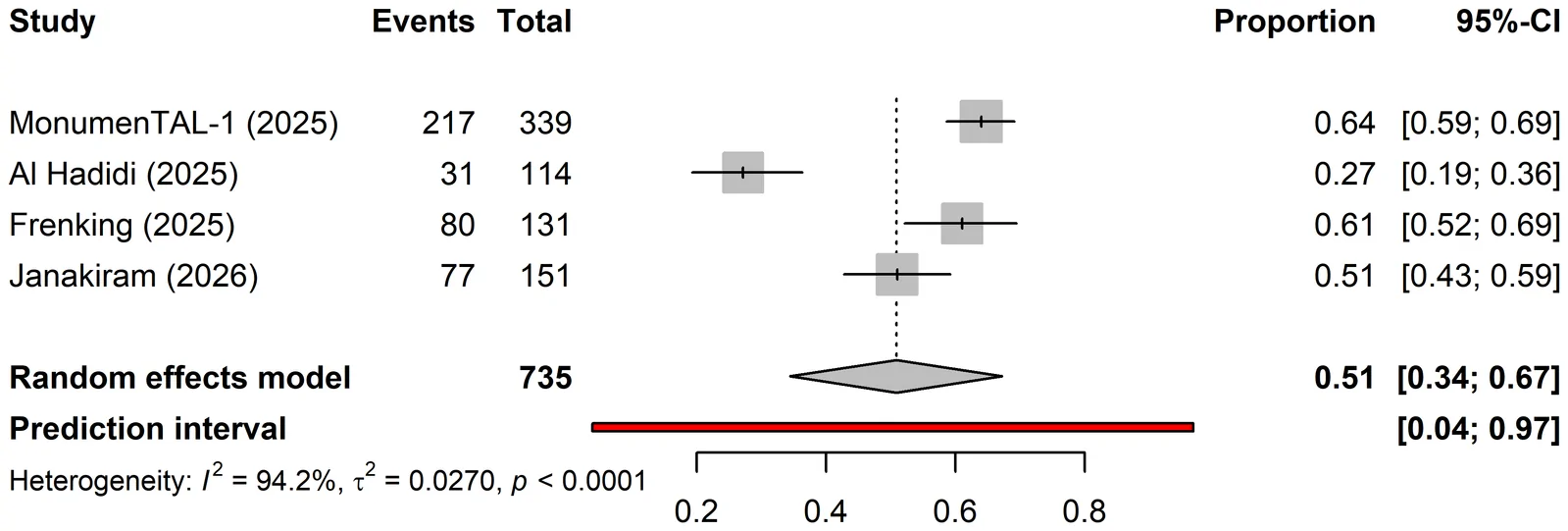
Forest plot of the pooled incidence of any-grade infections associated with talquetamab monotherapy. Forest plot showing the pooled incidence of any-grade infections among patients receiving talquetamab monotherapy. Four single-arm cohorts were included in the quantitative synthesis. The pooled incidence of any-grade infections was 51% (95% CI, 34%–67%) using a random-effects model. Substantial heterogeneity was observed among studies (I² = 94.2%, p < 0.001). Prediction intervals are presented to reflect between-study variability.

**Figure 3:**
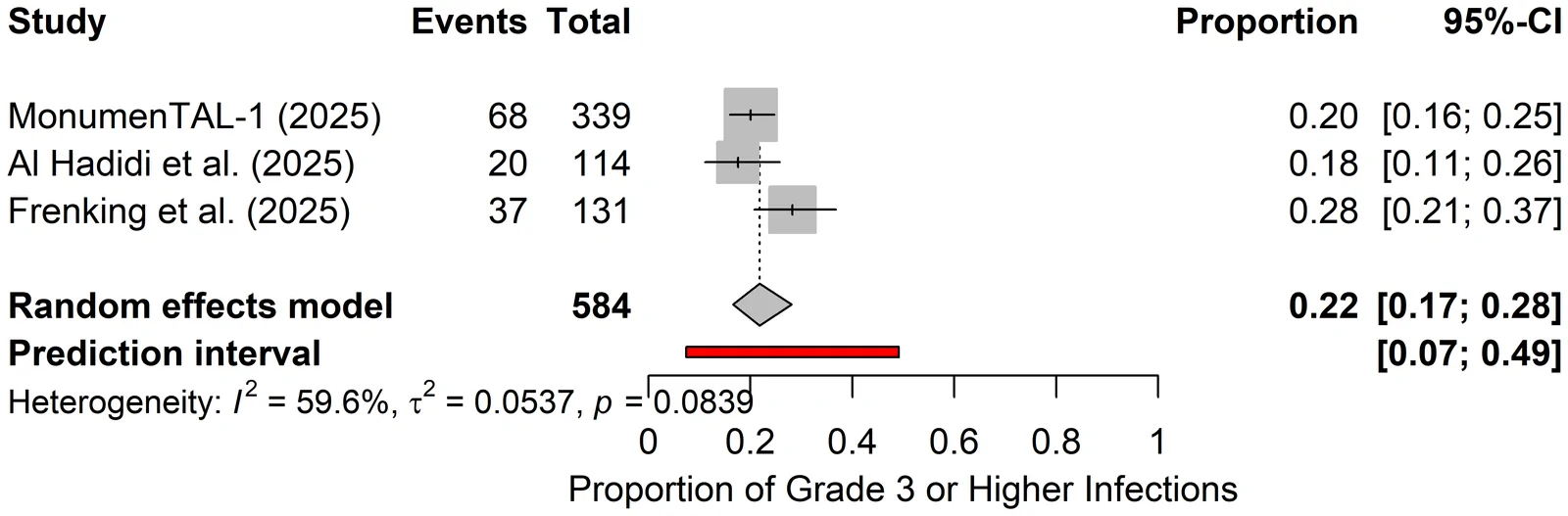
Forest plot of the pooled incidence of grade ≥3 infections associated with talquetamab monotherapy. Forest plot showing the pooled incidence of grade ≥3 infections among patients receiving talquetamab monotherapy. Three cohorts with available grade ≥3 infection data were included in the quantitative synthesis. The pooled incidence was 22% (95% CI, 17%–28%) using a random-effects model. Moderate heterogeneity was observed among studies (I² = 59.6%, p = 0.084). Prediction intervals are presented to indicate the expected range of true effects across different clinical settings.

Quantitative meta-analysis was restricted to talquetamab monotherapy cohorts. Infection outcomes from MonumenTAL-1 were extracted from the dedicated infection analysis report (25) rather than the primary efficacy publication (22). The pooled infection outcomes are summarized in **Table 2** and **Figures 2**, **3**.

**Table 2:** Summary of infection outcomes and infection characteristics across included studies.

Study	Treatment	Any-grade infection	Grade ≥3 infection	Infection-related mortality	Reported infection types/pathogens	IVIG use
MonumenTAL-1 (2025)	Talquetamab monotherapy	217/339 (64.0%)	68/339 (20.1%)	5/339 (1.5%)	Viral infections (including COVID-19); respiratory tract infections (including pneumonia); urinary tract infections; opportunistic viral and fungal infections	48/339 (14.2%)
Al Hadidi et al. (2025) (11)	Talquetamab monotherapy	31/114 (27.2%)	20/114 (17.5%)	0/114	Viral infections (COVID-19, rhinovirus); bacterial infections; opportunistic infections including Pneumocystis jirovecii pneumonia (PJP)	61%†
Frenking et al. (2025) (12)	Talquetamab monotherapy	80/131 (61.1%)	37/131 (28.2%)	NR	Bacterial, viral, and fungal infections; pathogens were not identified in some cases	69/138 (50.0%)
RedirecTT-1 (2025)	Talquetamab + teclistamab	84/94 (89.4%)	60/94 (63.8%)	3/94 (3.2%)	Viral infections (COVID-19, CMV, adenovirus); bacterial infections including pneumonia; opportunistic infections including PJP	NR
Janakiram et al. (2026) (23)	Talquetamab monotherapy	77/151 (51.0%)	37/151 (24.5%)	0/151	Upper respiratory infections; lung infections; bacteremia; urinary tract infections; viral reactivation including CMV reactivation; other unclassified infections	NR
MonumenTAL-3 (2026)	Talquetamab-based combination therapy (talquetamab + daratumumab ± pomalidomide)	492/574 (85.7%)‡	191/574 (33.3%)‡	6/574 (1.0%)‡	Viral infections (including COVID-19); bacterial infections including pneumonia; opportunistic infections including PJP; fungal infections	NR

CMV, cytomegalovirus; IVIG, intravenous immunoglobulin; NR, not reported; PJP, Pneumocystis jirovecii pneumonia.

Infection outcomes were extracted from the original reports. For MonumenTAL-1, infection outcomes were extracted from the dedicated infection analysis report, including 339 evaluable patients, whereas baseline characteristics were obtained from the updated primary report including 375 patients. Therefore, baseline characteristics and infection outcomes corresponded to different patient populations. For quantitative analyses, denominators were based on the evaluable populations reported in the corresponding infection-specific analyses rather than the overall enrolled populations when these differed.

In the Janakiram et al. cohort, infection severity grading was inconsistently reported. Severe infections requiring hospitalization (37/151 patients) were classified by the original authors as greater than grade 2 and are presented as a proxy for grade ≥3 infection. These data were summarized descriptively and were not considered equivalent to CTCAE-defined grade ≥3 infections for quantitative pooling.

^†^For the Al Hadidi et al. cohort, IVIG use was reported as 61%, although the exact number of patients receiving IVIG was not explicitly provided.

^‡^RedirecTT-1 and MonumenTAL-3 evaluated talquetamab-based combination regimens and were included for descriptive analysis only because these treatment strategies represented clinically distinct settings and were not included in quantitative pooling.

For Frenking et al., the overall cohort included 138 patients; however, infection outcomes were available for 131 evaluable patients. IVIG exposure was reported for the overall cohort.

Summary of infection-related outcomes among patients treated with talquetamab-containing regimens, including any-grade infections, grade ≥3 infections, infection-related mortality, reported pathogens, infection patterns, and intravenous immunoglobulin (IVIG) use when available. Quantitative synthesis was restricted to talquetamab monotherapy cohorts. Combination therapy cohorts, including RedirecTT-1 and MonumenTAL-3, were summarized descriptively because combined T-cell engager strategies may involve distinct immune suppression profiles and infection risks.

Any-grade infection: Four talquetamab monotherapy cohorts (MonumenTAL-1 infection analysis, Frenking et al., Al Hadidi et al., and Janakiram et al.) were included in the quantitative synthesis of any-grade infections (12, 22, 23, 25). The pooled incidence of any-grade infections was 51% (95% CI, 34%–67%; **Figure 2**). Considerable heterogeneity was observed among studies (I² = 94.2%, p < 0.001), indicating substantial variability in infection estimates across different clinical settings, patient populations, and supportive-care practices.

Grade ≥3 infection: Three cohorts with CTCAE-comparable grade ≥3 infection outcomes were included in the quantitative synthesis (12, 22, 25). The pooled incidence of grade ≥3 infections was 22% (95% CI, 17%–28%; **Figure 3**). Moderate heterogeneity was observed (I² = 59.6%, p = 0.084).

### 3.4 Descriptive analysis of talquetamab-based combination therapy

Talquetamab-based combination therapy cohorts were included in the qualitative synthesis only and were not incorporated into the quantitative meta-analysis because of potential differences in treatment intensity, immune effects, and infection risk profiles compared with talquetamab monotherapy.

Two prospective studies evaluating talquetamab-based combination regimens were identified. RedirecTT-1 (9) evaluated talquetamab plus teclistamab, whereas MonumenTAL-3 (24) evaluated talquetamab-based combination therapy with daratumumab with or without pomalidomide. These studies were analyzed descriptively because the combination regimens represented clinically distinct treatment strategies and could not be directly compared with talquetamab monotherapy cohorts.

In the RedirecTT-1 cohort (9), infections occurred in 84 of 94 patients (89.4%), including 60 patients (63.8%) with grade ≥3 infections. Reported infections included viral infections (including COVID-19, cytomegalovirus, and adenovirus), bacterial infections including pneumonia, and opportunistic infections such as Pneumocystis jirovecii pneumonia (PJP). Infection-related mortality occurred in 3 of 94 patients (3.2%).

In the MonumenTAL-3 study (24), infections occurred in 492 of 574 patients (85.7%), including 191 patients (33.3%) with grade ≥3 infections. Infection-related mortality was reported in 6 of 574 patients (1.0%). Reported infection types included viral infections (including COVID-19), bacterial infections including pneumonia, opportunistic infections including PJP, and fungal infections.

### 3.5 Sensitivity analysis

Leave-one-out sensitivity analyses were performed to evaluate the robustness of pooled estimates among talquetamab monotherapy cohorts. For both any-grade and grade ≥3 infections, sequential exclusion of individual studies resulted in broadly consistent pooled incidence estimates. However, individual cohorts materially influenced between-study heterogeneity. For example, exclusion of the Al Hadidi et al. cohort reduced heterogeneity for any-grade infections, whereas exclusion of the Frenking et al. cohort eliminated heterogeneity for grade ≥3 infections (**Supplementary Tables 4**, **S5**).

### 3.6 Infection-related mortality

Infection-related mortality data are summarized in **Table 2**. Infection-related deaths were reported inconsistently across included cohorts. In the MonumenTAL-1 infection analysis cohort (25), infection-related mortality occurred in 5 of 339 patients (1.5%). In the Al Hadidi et al. real-world cohort (11), no infection-related mortality was reported. Infection-related mortality was not reported in the Janakiram et al. cohort (23).

Among talquetamab-based combination therapy studies, RedirecTT-1 (9) reported infection-related mortality in 3 of 94 patients (3.2%), whereas MonumenTAL-3 (24) reported infection-related mortality in 6 of 574 patients (1.0%). These combination therapy data were summarized descriptively and were not included in the quantitative meta-analysis.

Reported severe and fatal infections included viral infections, particularly COVID-19, bacterial infections including pneumonia, and opportunistic infections such as Pneumocystis jirovecii pneumonia (PJP) and cytomegalovirus (CMV) infection (9, 12, 22–25). Differences in follow-up duration, infection definitions, surveillance strategies, prophylactic approaches, and supportive care may have contributed to variability in reported infection-related mortality across studies.

## 4 Discussion

In this systematic review and meta-analysis, we synthesized the available evidence regarding infection risk associated with talquetamab in patients with relapsed/refractory multiple myeloma (RRMM). Six studies were included in the qualitative synthesis, including four talquetamab monotherapy cohorts and two talquetamab-based combination therapy cohorts. Quantitative meta-analysis was restricted to talquetamab monotherapy cohorts, with four cohorts contributing data for any-grade infections and three cohorts contributing data for grade ≥3 infections. The pooled incidence of any-grade infections was 51%, and the pooled incidence of grade ≥3 infections was 22%, indicating a clinically meaningful burden of infections among heavily pretreated RRMM patients receiving talquetamab therapy. Although quantitative pooling was restricted to monotherapy cohorts, substantial heterogeneity remained, likely reflecting differences in patient characteristics, prior treatment exposure, follow-up duration, infection surveillance intensity, and supportive-care practices across clinical trial and real-world settings. Therefore, these pooled estimates should be interpreted as exploratory and hypothesis-generating given the limited number of available studies and variability in clinical contexts.

The substantial heterogeneity observed in the pooled estimates likely reflects multiple clinical and methodological factors. Patients included in the available studies were heavily pretreated, with high rates of triple-class refractory disease and previous exposure to BCMA-directed therapies (9, 11, 12, 22–24), suggesting substantial baseline immune dysfunction. Differences in patient selection, prior treatment burden, follow-up duration, infection surveillance intensity, infection definitions, and supportive-care practices may also have contributed to variability in reported infection rates. Furthermore, differences between prospective clinical trials and retrospective real-world studies, including adverse-event ascertainment and follow-up completeness, may also contribute to variability in reported infection rates (13, 14). Therefore, the observed heterogeneity likely reflects genuine differences in clinical populations and treatment environments rather than solely statistical variation. In addition, leave-one-out sensitivity analyses demonstrated that pooled point estimates remained broadly stable, although individual studies materially influenced between-study heterogeneity, indicating that variability was partly driven by specific cohort characteristics.

An important clinical implication of this analysis is the opportunity to better characterize the infection profile of GPRC5D-directed therapy in comparison with BCMA-targeted bispecific antibodies. Mechanistically, differences between these therapeutic classes are biologically plausible. Unlike BCMA, which is expressed on plasma cells and late-stage B-cell compartments, GPRC5D is expressed primarily on malignant plasma cells and certain keratinized tissues and is largely absent from early B-cell lineage precursors (26, 27). Therefore, GPRC5D-directed therapy may have a different impact on normal B-cell compartments and humoral immunity compared with BCMA-directed bispecific antibodies (26, 27). However, the substantial infection burden observed in our analysis indicates that mechanisms beyond hypogammaglobulinemia may also contribute to infectious susceptibility (28, 29). Additional contributing factors may include sustained T-cell activation, treatment-related cytopenias, cumulative prior immunosuppressive exposure, previous immune-directed therapies, and advanced disease-related immune dysfunction (28, 29). Supportive-care strategies, including immunoglobulin replacement, antimicrobial prophylaxis, and vaccination approaches, may further influence infection outcomes and warrant prospective investigation (25, 29).

Beyond overall infection incidence, the spectrum of infectious complications provides important insights into the clinical management of patients receiving talquetamab. Across included studies, infections involved viral, bacterial, and opportunistic pathogens, although pathogen reporting was heterogeneous across cohorts (9, 12, 22–25). Viral infections, particularly COVID-19, were frequently reported, while bacterial respiratory infections, including pneumonia, represented an important component of severe infectious complications (9, 12, 22–25). Opportunistic infections, including PJP and CMV infection, were also reported across included cohorts (9, 12, 22–25). These findings emphasize the importance of continued infection surveillance and early recognition of clinically significant infections in heavily pretreated RRMM patients receiving talquetamab therapy.

Preventive strategies during talquetamab therapy remain an area of clinical uncertainty. Supportive measures, including IVIG replacement, antimicrobial prophylaxis, vaccination, and prompt diagnostic evaluation, are commonly used in clinical practice, although their impact on infection outcomes remains uncertain. However, preventive interventions were inconsistently reported across studies, and differences in supportive-care practices may have contributed to heterogeneity in observed infection rates (9, 12, 22–25). IVIG utilization was reported in several cohorts, including the MonumenTAL-1 infection analysis (25), but the available evidence was insufficient to determine its association with infection prevention or reduction of severe infections. Future prospective studies should incorporate standardized reporting of immunoglobulin levels, IVIG exposure, antimicrobial prophylaxis, vaccination status, immune parameters, and infection outcomes to better define optimal prevention strategies.

The increasing use of talquetamab-based combination regimens represents an additional clinical consideration. Although RedirecTT-1 (9) and MonumenTAL-3 (24) reported substantial infection burdens, these findings should be interpreted cautiously because both studies evaluated treatment strategies that differ substantially from talquetamab monotherapy and were therefore not suitable for quantitative pooling. Future prospective studies directly comparing monotherapy and combination regimens are needed to better define the incremental infection risk associated with combination bispecific antibody therapy and to establish optimal preventive strategies.

This study has several strengths. It represents one of the first systematic evaluations focusing specifically on infection risk associated with talquetamab, incorporating evidence from both clinical trials and real-world studies. In addition, separating talquetamab monotherapy from combination regimens allowed more appropriate quantitative synthesis and avoided pooling clinically distinct treatment strategies. Furthermore, the inclusion of both pooled infection estimates and descriptive analyses of emerging combination regimens provides a more comprehensive overview of the evolving infection profile associated with talquetamab-based therapies.

Several limitations should be considered. First, the number of available studies remained limited, with only four monotherapy cohorts contributing to quantitative synthesis of any-grade infections and three cohorts contributing to grade ≥3 infection analysis. Therefore, pooled estimates should be interpreted as exploratory and hypothesis-generating. Publication bias could not be formally assessed because the number of included studies was insufficient for reliable evaluation.

Second, substantial heterogeneity was observed in pooled analyses, likely reflecting differences in patient characteristics, prior treatment exposure, follow-up duration, infection definitions, surveillance intensity, and supportive-care strategies. Although leave-one-out analyses demonstrated broadly stable pooled point estimates, individual cohorts materially influenced between-study heterogeneity.

Third, all quantitative analyses were based on uncontrolled single-arm cohorts without comparator groups, limiting the ability to determine the relative contribution of talquetamab therapy to infection risk compared with other treatment approaches. In addition, differences in study populations and treatment settings may limit direct comparisons across cohorts.

Finally, clinically relevant factors, including IVIG use, antimicrobial prophylaxis, vaccination status, immunoglobulin levels, and other immune parameters, were inconsistently reported across studies, preventing a robust assessment of their impact on infection outcomes. Future prospective studies with standardized reporting of infection outcomes and preventive strategies are needed.

In conclusion, this systematic review and meta-analysis suggests that talquetamab monotherapy is associated with a clinically meaningful burden of infections, including severe infectious complications, among heavily pretreated patients with RRMM. The observed infection spectrum included viral, bacterial, and opportunistic infections, highlighting the need for continued infection surveillance in patients receiving talquetamab therapy. Although talquetamab-based combination regimens were not included in quantitative pooling, descriptive analyses of RedirecTT-1 and MonumenTAL-3 reported substantial infection burdens in these emerging treatment settings. However, the limited number of available studies, substantial heterogeneity, and absence of comparator groups restrict definitive conclusions. Further prospective studies with standardized reporting of infection outcomes, immune parameters, and preventive strategies are needed to better define optimal infection prevention and management strategies as talquetamab-based therapies continue to expand in clinical practice.

## Supplementary material

Supplementary Table 1 PICOS framework defining eligibility criteria for study selection. The table summarizes the Population, Intervention, Comparator, Outcomes, and Study design criteria used for study eligibility assessment. Eligible studies included adults with relapsed/refractory multiple myeloma receiving talquetamab-containing therapy and reporting infection-related outcomes.

Supplementary Table 2 Database-specific search strategies and search dates. Detailed search strategies and search dates for PubMed, Embase, Web of Science Core Collection, and Cochrane Library. All databases were searched from inception to July 1, 2026. Search strategies were adapted according to database-specific requirements.

Supplementary Table 3 Methodological quality assessment of included single-arm cohorts using the Joanna Briggs Institute Critical Appraisal Checklist for Case Series. Detailed methodological quality assessment of four single-arm cohorts included in quantitative synthesis using the JBI Critical Appraisal Checklist for Case Series. Most domains were rated as “Yes,” indicating generally acceptable methodological quality. Domains with insufficient reporting were rated as “Unclear.” Although MonumenTAL-1 originated from a prospective clinical trial, infection outcomes were extracted from an uncontrolled single-arm safety cohort; therefore, the case-series framework was considered appropriate for evaluating methodological limitations related to infection reporting.

Supplementary Table 4 Leave-one-out sensitivity analysis of the pooled incidence of any-grade infections associated with talquetamab monotherapy. Sequential leave-one-out sensitivity analyses were performed by removing each individual monotherapy cohort from the meta-analysis. The pooled incidence estimates remained broadly consistent after exclusion of individual studies; however, heterogeneity estimates varied depending on the omitted cohort, indicating that individual studies contributed differently to between-study variability.

Supplementary Table 5 Leave-one-out sensitivity analysis of the pooled incidence of grade ≥3 infections associated with talquetamab monotherapy. Sensitivity analyses were conducted by sequential exclusion of individual studies evaluating grade ≥3 infections. The pooled estimates remained broadly stable after omission of individual cohorts; however, exclusion of certain studies influenced between-study heterogeneity, suggesting differential contributions to variability across studies.

Supplementary Table 6 Full-text articles excluded after eligibility assessment with reasons for exclusion. The table lists studies excluded after full-text review and provides specific reasons for exclusion, including review/editorial articles, lack of extractable infection outcomes, preclinical or case report studies, overlapping cohorts, and inappropriate populations or interventions.

Supplementary Figure 1 Methodological quality assessment summary of included studies using the Joanna Briggs Institute Critical Appraisal Checklist for Case Series. Heatmap visualization showing methodological quality assessments across ten JBI domains for the four single-arm cohorts included in quantitative synthesis. Green indicates “Yes,” whereas yellow indicates “Unclear.” The figure summarizes the methodological quality profile of included studies.
