# Supplementary material for: Infection risk associated with talquetamab in relapsed/refractory multiple myeloma: a systematic review with meta-analysis of talquetamab monotherapy and descriptive analysis of combination therapy
Source: Front Immunol. 2026 Aug 19;17:1848518. doi: 10.3389/fimmu.2026.1848518 (PMC13534137; doi:10.3389/fimmu.2026.1848518)
Supplement: Supplementary file 1 [file SupplementaryFile1.docx]

**Supplementary Table S1. PICOS framework defining the eligibility criteria for study selection.**

| **PICOS element** | **Eligibility criteria** |
| --- | --- |
| **Population (P)** | Adult patients (≥18 years) with previously treated relapsed or refractory multiple myeloma (RRMM) receiving talquetamab-containing therapy. |
| **Intervention (I)** | Talquetamab-containing therapy, including talquetamab monotherapy or combination regimens. Studies evaluating combination therapies were eligible but were analyzed separately because of potential differences in treatment-related immune effects. |
| **Comparator (C)** | Not applicable. This review synthesized single-arm cohorts; therefore, no comparator group was required. |
| **Outcomes (O)** | Infection-related outcomes, including any-grade infections, grade ≥3 infections, infection-related mortality, reported infection types/pathogens, and preventive strategies such as intravenous immunoglobulin (IVIG) use when available. |
| **Study design (S)** | Prospective clinical trials and observational real-world studies reporting infection outcomes among patients treated with talquetamab-containing regimens. |

### **Supplementary Table S2. Database-specific search strategies and search dates**

| Database | Search date | Search strategy | Records identified |
| --- | --- | --- | --- |
| **PubMed (MEDLINE)** | July 1, 2026 | ("talquetamab"[Title/Abstract] OR "GPRC5D"[Title/Abstract] OR "JNJ-64407564"[Title/Abstract] OR "JNJ64407564"[Title/Abstract] OR "Talvey"[Title/Abstract]) AND (infection[Title/Abstract] OR infectious[Title/Abstract] OR pneumonia[Title/Abstract] OR sepsis[Title/Abstract] OR COVID-19[Title/Abstract] OR viral[Title/Abstract] OR bacterial[Title/Abstract] OR fungal[Title/Abstract] OR opportunistic[Title/Abstract] OR "adverse event"[Title/Abstract]) | **52** |
| **Embase** | July 1, 2026 | ('talquetamab':ti,ab OR 'gprc5d':ti,ab OR 'jnj-64407564':ti,ab OR 'jnj64407564':ti,ab OR 'talvey':ti,ab) AND ('infection'/exp OR infection:ti,ab OR infectious:ti,ab OR pneumonia:ti,ab OR sepsis:ti,ab OR 'covid-19':ti,ab OR viral:ti,ab OR bacterial:ti,ab OR fungal:ti,ab OR opportunistic:ti,ab OR 'adverse event':ti,ab) | **319** |
| **Web of Science Core Collection** | July 1, 2026 | TS=(talquetamab OR GPRC5D OR JNJ-64407564 OR JNJ64407564 OR Talvey) AND TS=(infection OR infectious OR pneumonia OR sepsis OR COVID-19 OR viral OR bacterial OR fungal OR opportunistic OR "adverse event") | 31 |
| **Cochrane Library** | July 1, 2026 | (talquetamab OR GPRC5D OR JNJ-64407564 OR JNJ64407564 OR Talvey) AND (infection OR infectious OR pneumonia OR sepsis OR COVID-19 OR viral OR bacterial OR fungal OR opportunistic OR "adverse event") | **14** |

Notes: All databases were searched from inception to July 1, 2026. No language restrictions were applied during database searching; however, eligible studies were restricted to English-language publications during screening. Search strategies were adapted according to the requirements of each database interface. Duplicate records were removed before title and abstract screening.

**Supplementary Table S3. Methodological quality assessment of studies included in quantitative synthesis using the Joanna Briggs Institute Critical Appraisal Checklist for Case Series.**

| **Study** | **Q1** | **Q2** | **Q3** | **Q4** | **Q5** | **Q6** | **Q7** | **Q8** | **Q9** | **Q10** |
| --- | --- | --- | --- | --- | --- | --- | --- | --- | --- | --- |
| MonumenTAL-1 (2025) | Yes | Yes | Yes | Unclear | Yes | Yes | Yes | Yes | Unclear | Yes |
| Al Hadidi et al. (2025) | Yes | Yes | Yes | Unclear | Unclear | Yes | Yes | Yes | Unclear | Yes |
| Frenking et al. (2025) | Yes | Yes | Yes | Unclear | Unclear | Yes | Yes | Yes | Unclear | Yes |
| Janakiram et al. (2026) | Yes | Yes | Yes | Unclear | Unclear | Yes | Yes | Yes | Unclear | Yes |

****Notes:**** The Joanna Briggs Institute (JBI) Critical Appraisal Checklist for Case Series was applied to studies included in the quantitative synthesis. Although MonumenTAL-1 originated from a prospective clinical trial, infection outcomes were extracted from an uncontrolled single-arm safety cohort without a comparative control group; therefore, the case-series framework was considered appropriate for assessing methodological limitations related to infection reporting. “Unclear” indicates that relevant methodological information was insufficiently reported.

**Supplementary Figure S1. Quality assessment of included studies using the Joanna Briggs Institute Critical Appraisal Checklist for Case Series.**

****Notes:**** Green indicates "Yes" and yellow indicates "Unclear". Q1–Q10 represent the ten domains of the JBI Critical Appraisal Checklist for Case Series.

**Supplementary Table S4. Leave-one-out sensitivity analysis for the pooled incidence of any-grade infections associated with talquetamab monotherapy.**

| **Omitted study** | **Pooled incidence (95% CI)** | **Heterogeneity (I², %)** |
| --- | --- | --- |
| None (overall) | 0.51 (0.34–0.67) | 94.2% |
| MonumenTAL-1 (2025) | 0.47 (0.42–0.52) | 93.5% |
| Al Hadidi et al. (2025) | 0.60 (0.56–0.64) | 72.6% |
| Frenking et al. (2025) | 0.54 (0.50–0.58) | 95.9% |
| Janakiram et al. (2026) | 0.56 (0.52–0.60) | 96.0% |

**Notes:** Each analysis was repeated after sequential exclusion of one individual monotherapy cohort. The pooled estimates remained generally consistent after omission of individual studies, suggesting that no single study had a disproportionate influence on the overall pooled incidence.

**Supplementary Table S5. Leave-one-out sensitivity analysis for the pooled incidence of grade ≥3 infections associated with talquetamab monotherapy.**

| Omitted study | Pooled incidence (95% CI) | Heterogeneity (I², %) |
| --- | --- | --- |
| None (overall) | 0.22 (0.17–0.28) | 59.6% |
| MonumenTAL-1 (2025) | 0.23 (0.14–0.35) | 74.0% |
| Al Hadidi et al. (2025) | 0.24 (0.17–0.32) | 72.3% |
| Frenking et al. (2025) | 0.20 (0.16–0.23) | 0% |

Notes: Each analysis was repeated after sequential exclusion of one individual study. The pooled estimates remained generally consistent, suggesting that no single study substantially influenced the overall findings.

**Supplementary Table S6. Full-text articles excluded after eligibility assessment with reasons for exclusion.**

| First author | Year | Study/article | Reason for exclusion |
| --- | --- | --- | --- |
| Verkleij | 2021 | Preclinical activity and determinants of response of the GPRC5D×CD3 bispecific antibody talquetamab | Preclinical study; no clinical patient population or infection outcomes |
| Labanca | 2025 | Talquetamab in Multiple Myeloma: Efficacy, Safety, and Future Directions | Review article; no original infection-related data |
| Moore | 2025 | Talquetamab for the treatment of relapsed/refractory multiple myeloma: efficacy, safety, and real-world evidence | Review article; no independent clinical cohort |
| Tariq | 2023 | Talquetamab: A promising immunotherapy for multiple myeloma | Narrative review; no extractable infection outcomes |
| Miller | 2025 | Talquetamab: Emerging role in multiple myeloma | Review article; no original infection dataset |
| Keam | 2023 | Talquetamab: First approval | Drug approval review; no original clinical infection data |
| Shaver | 2025 | Targeting GPRC5D With Talquetamab: A New Frontier in Bispecific Antibody Therapy for RRMM | Narrative review; no independent patient-level infection data |
| Chari | 2024 | Clinical management of patients with RRMM treated with talquetamab | Management/review article; no extractable infection incidence |
| Sandahl | 2025 | Management of patients with RRMM treated with talquetamab: highlights from pharmacists' perspectives | Educational review; no original clinical data |
| Matsumura | 2026 | Regulatory update regarding talquetamab-containing therapy | Regulatory report/editorial; no original infection outcomes |
| Alves | 2026 | On-target, off-tumor oral toxicities of talquetamab | Focused on non-infectious toxicity; no infection outcomes |
| Heini | 2024 | Recurrent talquetamab skin toxicity after supportive stem cell boost | Case report; no population-level infection data |
| Zayad | 2024 | Dermatological adverse events linked to talquetamab immunotherapy | Case report; no extractable infection outcomes |
| Dhakal | 2025 | Talquetamab as a bridge to BCMA CAR-T therapy | Infection outcomes were related mainly to subsequent CAR-T therapy; talquetamab-specific infection data were unavailable. |
| Hamadeh | 2026 | Defining the rates of cytokine release syndrome associated with talquetamab step-up doses | Study focused on CRS; no infection outcomes |
| Di Palma-Grisi | 2026 | Salvage therapy with talquetamab, elranatamab or teclistamab in combination with pomalidomide | Combination T-cell engager therapy; talquetamab-specific infection outcomes not separately extractable |
| Ito | 2026 | Talquetamab in Japanese Patients with Relapsed/Refractory Multiple Myeloma in the MonumenTAL-1 Study | Companion analysis from the MonumenTAL-1 clinical trial program; excluded as an independent cohort because potential overlap with the primary MonumenTAL-1 infection analysis population could not be excluded. |
| Kumar | 2026 | Talquetamab-based therapy for extramedullary myeloma | Dedicated analysis of an extramedullary disease subgroup; excluded because the population represented a selected high-risk subgroup and was not considered an independent cohort for the predefined overall RRMM synthesis. |

**Note:** Publications originating from the same clinical trial population were considered companion reports and were not counted as independent studies. The dedicated infection analysis of the MonumenTAL-1 cohort was used for extraction of infection-related outcomes but was not considered an additional independent study.
